# Oxidized Hyaluronic Acid Hydrogels as a Carrier for Constant-Release Clenbuterol Against High-Fat Diet-Induced Obesity in Mice

**DOI:** 10.3389/fendo.2021.572690

**Published:** 2021-03-12

**Authors:** Wei-Yao Chen, Feng-Huei Lin

**Affiliations:** ^1^Institute of Biotechnology, National Taiwan University, Taipei, Taiwan; ^2^Institute of Biomedical Engineering, National Taiwan University, Taipei, Taiwan; ^3^Institute of Biomedical Engineering and Nanomedicine, National Health Research Institutes, Miaoli County, Taiwan

**Keywords:** obesity, clenbuterol, abuse, constant release, hydrogel

## Abstract

The global obesity population is increasing year-by-year, and the related cost is sharply increasing annually. There are several methods available to combat obesity; however, there is a lack of a single tool that is both safe and efficacious. The use of Clenbuterol in bodybuilding and by professional athletes is controversial owing to its side effects, including hepatotoxicity. This study administered Clenbuterol at a much lower dose than the established safety level, and rather than through oral administration, the treatments were delivered through controlled-release intra-adipose injection. The different dosing and mode of administration will lower the risk of side effects, increase the safety profile, and could facilitate use in the anti-obesity market. A thermo-sensitive hydrogel was used as the carrier uploaded with Clenbuterol to achieve controlled-release. In the *in vitro* study, the developed new formulae were not cytotoxic to 3T3-L1 cells and could inhibit lipogenesis effectively. In the animal study, the mice were fed a high-fat diet and treated with Clenbuterol by oral administration, or injected with Clenbuterol-modified hyaluronate hydrogel (HAC) regularly. Both groups showed reduction in whole-body, visceral, and gonadal fat contents and body weight. The abdominal fat was analyzed using MRI imaging in adipose mode and water mode. The abdominal fat ratio in the mice treated with normal diet and those given intra-adipose injections with HAC had the lowest value among the test groups. The mice treated with high-fat diet (HFD) showed the highest value of 53.78%. The chronic toxicity *in-vivo* test proved that controlled-release injections of 2–10 µg Clenbuterol daily were safe, as demonstrated in the blood elements and serological analyses. This study developed a new and promising method for anti-obesity treatment, using a monthly intra-adipose controlled-release injection of HAC. The developed new formulae of Clenbuterol not only effectively decreased body weight and body fat content but also inhibited lipogenesis on the harvested visceral tissue and reduced adipose tissue around the gonadal fat area. The side effects induced by traditional oral administration of Clenbuterol were not observed in this research; this has excellent potential to be a useful tool for future obesity treatment without safety concerns.

## Introduction

Obesity arises from imbalance of food intake and calorie expenditure. The standard way of classifying individuals’ weights into underweight, normal weight, overweight, or obese is based on the ratio of body weight and the square of the body height, called the body mass index (BMI) (1). In modern society, the population identified as obese is increasing in number yearly, which may be related to many diseases, such as diabetes, high blood pressure, heart disease, gallbladder disease, gallstones, and osteoarthritis. According to statistics, about 2.8 million people die each year related to obesity, and the global cost of obesity-related medical care is up to $ 2.0 trillion annually. The excessive consumption of unhealthy high-fat dietary, such as low-quality oil, sugary drinks and salty snacks are usually significant causes of obesity (2).

Several methods are available to combat obesity, for instance, exercise, dietary control, functional foods, daily supplements, pharmaceutical treatments, and surgery. Physical activity is the most convenient method to burn calories. However, people are generally not able to sustain long-term and regular exercise. Several dietary supplements with alginate-based, chitosan-based, herb-based, vitamins, or minerals have been developed and are available on the market for use by individuals to control body weight (3–5). Although some showed promising results, they lacked sufficient efficacy. The pharmaceutical treatments such as Orlistat (Xenical), Lorcaserin (Belviq^®^) liraglutide, phentermine/topiramate, and naltrexone/bupropion, are effective agents against obesity for long-term (6). However, the pharmacodynamic process of the digested drug might induce side effects owing to metabolic disequilibrium, for instance, headache, dizziness, back pain, flatulence, abdominal pain or discomfort, and inability to control stool (incontinence). Many people look for more invasive ways to combat obesity using bariatric surgery methods, such as gastrectomy, enterectomy, or usage of biometrics intragastric balloon. However, these are expensive and have long recovery times post-operation (7, 8). We, therefore, believe that there is no one method currently available that fully satisfies customers’ requirements for both safe and efficacious obesity treatment.

Clenbuterol, a sympathomimetic amine, was developed initially to treat asthma in horses and then as an asthma drug for humans in some European and Latin American countries. It was approved in 1991 by U.S. Food Safety and Inspection Service for use in animals to increase the ratio of fat/lean mass in muscle tissue. Subsequent studies found that it induced hepato-toxicity and so was excluded from human use in Canada and the USA. Orally administered Clenbuterol is rapidly digested and metabolized into inactive compounds by the liver before it reaches systemic circulation; 4-N-Hydroxylamine is the major metabolite detected in rat’s urine. Clenbuterol hepatotoxicity is a side effect that occurs as a result of oral intake and first-pass metabolism. Other side effects of Clenbuterol include increased heart rate, rapid breathing, heart palpitations, chest pain, tremors, anxiety, and electrolyte imbalance (9). Although controversial, Clenbuterol has been identified as an effective non-steroidal hormone for muscle-mass synthesis and obesity treatment. It is still regularly used by many bodybuilding and professional athletes, even with the risk of body destruction and the early suspension of their professional career (8). The daily safe oral dose range of Clenbuterol for acute asthma is approximately 20-40 micrograms (10–12). We do believe the safety concern can be addressed through the administration of Clenbuterol at a dose far less than the established safety range. It can also be delivered by controlled-release intra-adipose injection rather than oral administration. These alternate methods may decrease the previously mentioned side effects allowing for safe usage in the anti-obesity market. In the study, we are going to use a thermo-sensitive hydrogel as the carrier uploaded with Clenbuterol to achieve controlled-release.

Hyaluronate (HA) is a biocompatible, non-toxic, and biodegradable biopolymer; it is the major component in most of the extracellular matrix (ECM) and is one of the earliest ECMs that appear in the embryonic stage. HA is actively involved in the regulation of cell division, migration, differentiation, and tissue/and organ regeneration at all stages of organism development or ontogenesis (13). It has been widely used in treatments such as orthopedics, dentistry, plastic surgery, skin ulcer, wound dressing, drug/gene/cell carrier, and anti-adhesion post-surgery (14).

In the study, Clenbuterol and hyaluronate (HA) would be oxidized by sodium periodate (NaIO_4_) to create a highly reactive ketone group and two aldehyde groups, respectively; abbreviated as oxi-Clenbuterol and oxi-HA. The oxi-Clenbuterol and oxi-HA would be mixed with, and crosslinked by, adipic acid di-hydrazide (ADH) to turn them into a thermo-sensitive hydrogel (Clenbuterol-modified hyaluronate hydrogel, HAC). Clenbuterol would be grafted on the molecular chain of HA for constant release. The formula design would be a once a month single dose given by controlled-release intra-adipose injection for future use in anti-obesity clinics (15).

The HAC hydrogel was prepared and then checked using Fourier-transform infrared spectroscopy (FTIR), Nuclear Magnetic Resonance (NMR), and rheometer to confirm functional groups, molecular structure, and gelation time and temperature, respectively. The release profile *in vitro* was used to soak HAC in PBS solution for some time and then check the Clenbuterol level in the supernatant by UV-Vis. Those comprehensive data were previously published in the material-related journal (15–18). In the manuscript, we focused more on the results of the following: cell viability, cytotoxicity, lipolysis on a cellular level, chronic toxicity, body weight control, whole-body adipose tissue, gonadal fat tissue, blood analysis, serological analysis, and sectioning examination of internal organs. These would be used to evaluate the safety and efficacy of the synthesized HAC, both *in vitro* and *in vivo*.

Cell viability and cytotoxicity would be checked using a WST-1 and LDH assay, respectively. The Oil-Red assay was used to first screen the effectiveness of HAC on lipolysis *in vitro* using 3T3-L1. A 4-week old C57BL/6 male mouse (average weight 20 g) would be used as an experimental animal. The mouse was induced to be obese through a high-fat diet. The synthesized HAC hydrogel was injected into the left side of the gonad fat tissue, the most common area to evaluate waist fat lipolysis and adipogenesis of the mouse, and the right side was kept as a reference. The injection administration was conducted once a month for 22 weeks. The body weight was measured once a week using an electronic scale. Before being sacrificed, the area of adipose tissue in the abdomen was measured using the magnetic resonance nuclear (MRI) with a cross-section image of the abdomen. After being sacrificed, the whole-body fat was harvested and weighed using an electronic scale. Gonadal adipose tissue was selected to compare the fat weight on both sides of the gonad adipose tissue with/without HAC injection as parallel-evidence for the efficacy of HAC on lipolysis *in vivo*. HAC sub-chronic toxicity was then evaluated using blood element analysis, serological analysis, and hematoxylin-eosin (HE) staining of all the organs. Clenbuterol by oral administration would also be used as a control group to compare with the HAC experimental group.

## Materials and Methods

### The Preparation of Injectable Clenbuterol-Modified Hyaluronate Hydrogel (HAC)

The major reagents used to synthesize HAC hydrogel were Clenbuterol, hyaluronate (HA), and adipic acid di-hydrazide (ADH). Clenbuterol was purchased from Sigma-Aldrich, Inc. (St. Louis, MO, USA) C5423, Lot#BCBF1701V. HA (1.9 × 10^6^ Da average molecular weight) was obtained from Sigma-Aldrich, Inc. (St. Louis, MO, USA) 53747, Lot# BCBX0145. ADH was purchased from Sigma-Aldrich, Inc. (St. Louis, MO, USA) A0638, Lot# SLBW1095. NaIO_4_ was purchased from Sigma-Aldrich, Inc. (St. Louis, MO, USA) S1878, Lot# MKBD4647V.

The preparation of injectable HAC hydrogel was based on the protocol reported by previous studies with minor modifications (19). Firstly, Clenbuterol was oxidized using NaIO_4_, oxi-Clenbuterol, to create a highly reactive ketone group that would be used to link to the HA chain *via* ADH in a later experiment.

1 mM Clenbuterol solution was prepared and placed into Eppendorf by 2 ml. 0.15 M of NaIO_4_ solution was made and added into the previous Eppendorf by 4 µl, well-mixed by shaking. 1 g HA was dissolved in 100 ml of dd-H_2_O and then oxidized by NaIO_4_, oxi-HA, to create two aldehyde groups at 2^nd^ and 3^rd^ carbon of HA 6-membrane ring; and followed by vacuum drying and stored in -20°C for later use. 0.5 g of ADH was dissolved in 5 ml of PBS. 6 g oxi-HA was dissolved in 100 ml of PBS and then added into 60 ml of ADH solution (oxi-HA-ADH). Finally, an adequate amount of oxi-Clenbuterol was added to complete the synthesis of injectable HAC thermo-sensitive hydrogel (15–18, 20).

### Cell Culture and Differentiation

3T3-L1 pre-adipocytes were purchased from the Bioresource Collection and Research Center (BCRC). Dulbecco’s modified Eagle’s medium (DMEM) - high glucose was obtained from Gibco (Waltham, Thermo Fisher Scientific, USA) 11965092. FBS was bought from Gibco (Waltham, Thermo Fisher Scientific, USA). IBMX was bought from Sigma-Aldrich, Inc. (St. Louis, MO, USA) I5879. Dexamethasone was obtained from Sigma-Aldrich, Inc. (St. Louis, MO, USA) D2915, Lot#031M1358V. Insulin was purchased from Sigma-Aldrich, Inc. (St. Louis, MO, USA) DIF001A.

3T3-L1 pre-adipocytes were cultured in DMEM - high glucose supplemented with 10% fetal bovine serum (FBS-DMEM) at 37°C in a 5% CO_2_ humidified atmosphere. For adipocyte differentiation, two-day post-confluent cells were placed in 10% FBS-DMEM with 250 nM dexamethasone, 0.5 mM IBMX, and insulin (1 μg/ml). After cultured for two days, the cells were cultured in 10% FBS-DMEM containing insulin (1 μg/ml) for two more days to induce cells toward adipogenesis. The cells were then called 3T3-L1 adipocytes and maintained in 10% FBS-DMEM for later experiments.

### Cell Viability and Cytotoxicity

WST-1 kit was obtained from Roche, Inc. (Basel, Switzerland) 05015944001 and used to evaluate cell viability. T

The 3T3-L1 adipocytes were seeded onto a 96-well culture dish at a density of 3×10^3^ cells/well for later tests. The test groups are described in **Table 1** as follows: C (control) group, OC (oxidize Clenbuterol) group, HA (oxi-HA crosslinked by ADH) group, and HAC (oxi-Clenbuterol grafted on ADH-crosslinked oxi-HA) group.

**Table 1:** The description of abbreviation for each group on *in vitro* study.

Abbreviation	C	OC	HA	HAC
Description	Non-treatment as Blank Control	10 ^-8^ M oxi-Clenbuterol	8% ADH-crosslinked 6% oxi-HA	10 ^-8^ M oxi-Clenbuterol grafted on 8% ADH-crosslinked 6% oxi-HA

After a one-day culture of 3T3-L1 adipocytes, the 200 µl of FBS-DMEM were added into each well and then added in the OC, HA, or HAC. The control group was defined as 3T3-L1 adipocytes cultured in 200 µl of FBS-DMEM only. After another one-day culture, the medium was replaced by the WST-1 kit mixed with FBS-DMEM by volume ratio of 1:1 wrapped in Sn-foil and then placed in an incubator to react for 30 min. The medium was collected to analyze in ELISA reader at a wavelength of 450 nm. The 3^rd^-day data was obtained using the same previously mentioned protocol.

Cytotoxicity was evaluated using an LDH assay (CytoTox 96^®^ Non-Radioactive Cytotoxicity Assay) that was purchased from Promega (Madison, WI, USA) G1780. The cell preparation was completed similarly to WST-1. The abbreviation and description of the test groups are described in **Table 1**. For cytotoxicity assay, lactate dehydrogenase (LDH) assay was performed according to the manufacturer’s instructions. LDH released in the medium was quantitatively assessed by spectrophotometer at 490 nm.

Percentage of cytotoxicity was expressed using the following formulae:

(1) $$\text{Cytotoxicity}\;(\%)=\frac{\text{Medium}\;\text{O.D}-\text{Blank}\;\text{O.D}}{\text{Total}\;\text{Lysis}\;\text{O.D.}-\text{Blank}\;\text{O.D.}}\times 100\%$$

### Oil-Red-O

Oil-Red-O was used to check the oil-drop formation in the cytoplasm of the 3T3-L1 adipocyte; the kit was obtained from Sigma-Aldrich, Inc. (St. Louis, MO, USA) 13200605. The cell preparation was similar to that for WST-1. The test groups are described in **Table 1**.

3T3-L1 adipocytes were cultured at least for three days to stabilize the adipocyte differentiation. They were then washed with phosphate-buffered saline (PBS) and fixed with 4% paraformaldehyde in 0.1 M phosphate buffer (pH 7.4) for 10 min at room temperature. The cells were washed two times with dd-H_2_O. A mixture of Oil Red O (0.5% Oil-Red-O dye in isopropanol) and water in a 6:4 ratio was layered on the cells for 30 min, followed by washing two times with dd-H_2_O. The culture dish was mounted on the microscope to examine the oil-drop formation in the cytoplasm. The oil-drop formation was quantified using an ELISA reader at 520 nm wavelength.

### Animal Study

The 4-week-old C57BL/6 male mice were purchased from BioLASCO Taiwan Co., Ltd. The mice were kept at Laboratory Animal Center, College of Medicine, National Taiwan University, accredited by Association for Assessment and Accreditation of Laboratory Animal Care (AAALAC), for 2 weeks before the experiment. The mice were randomly distributed as five per polycarbonate cage in a well-controlled environment, where light switched on at 08:00 and off at 20:00 h, at the temperature of 25 ± 2°C and relative humidity of 50 ± 10%.

6-week-old C57BL/6 male mice were fed a high-fat diet (HFD) containing 60% energy as fat, purchased from BioLASCO Taiwan Co., Ltd, DYET#101920, to induce obesity (21–23).

The mice were divided into five groups, with five mice in each group (n = 5). The five groups were briefly described as follows:

(1) The mice fed with normal diet were assigned as the control group (ND); (2) The mice fed with high-fat diet served as obesity mice (HFD); (3) The mice fed with a HFD and orally-administered Clenbuterol followed by **Table 1** were the contrast group (C-oral). The dosage in drinking water is 0.05 mg/kg - 0.20 mg/kg daily for 2 weeks each month (during the HAC constant release); (4) The mice fed with HFD and injected with ADH-crosslinked oxi-HA group (HA) (5) The mice fed with HFD and injected with 0.1 µl of HAC hydrogel in the gonad adipose tissue once a month were assigned as the experimental group (HAC). Food intake data of all group showed in **Supplementary Figure 2**.

### Magnetic Resonance Imaging (MRI)

The MRI used in the study was Bruker BioSpec 70/30 MRI 7T located at R134 Interdisciplinary Magnetic Resonance Lab, Department of Electrical Engineering, National Taiwan University; it was used for adipose tissue analysis. Chemical-shift imaging could be used to accurately decompose water and fat signals from the acquired MRI image. A proton density fat fraction (PDFF) could be calculated from the separated images and reflected the relative fat content by a voxel-by-voxel basis. The PDFF is mathematically related to the fat mass fraction closely and could be converted to absolute fat mass in grams by multiplying by the voxel volume and the mass density of fat (24). The MRI was performed using the following parameters: RARE-T1, averages 4, TR 1935.7ms, TE 9.0ms, Fov 4x4, MTX 256x256, thickness 0.5 mm (Vertical Section), 1 mm (horizontal Section) for increased precision (25).

### The Calculation of Body Weight Gain

The weight gain of the experimental mice was calculated by the following formulae

(2) $$\text{The}\;\text{percentage}\;\text{of}\;\text{weight}\;\text{gain}\;(\%)=\frac{{\text{W}}_{\text{S}}-{\text{W}}_{\text{O}}}{{\text{W}}_{\text{O}}}\times 100\%$$

Ws was the bodyweight of the mouse before being sacrificed, and Wo was the bodyweight of the mouse at the start of the experiment

### Percentage of Body Fat

We collected adipose tissue from the visceral fat (epididymal, mesentery, perirenal, retroperitoneal adipose tissue) and subcutaneous fat (anterior, posterior, dorsolumbar, inguinal, gluteal adipose tissue).

The percentage of body fat was calculated as the following formula:

(3) $$\text{The}\;\text{percentage}\;\text{of}\;\text{whole}\;-\text{body}\;\text{fat}\;(\%)=\frac{\text{TWBF}}{\text{BW}}\times 100\%$$

TWBF is the abbreviation for total weight of the body fat, which is the fat tissue collected from the previous tissues, and BW is the abbreviation for bodyweight of the mouse. The unit of the weight in the formulae (3) is all in gram (g).

The visceral fat was defined as the fat tissue collected from epididymal, mesentery, perirenal, retroperitoneal fat pad, used to evaluate the quantity of the “active fat.” It was calculated using the following formula:

(4) $$\text{The}\;\text{percentage}\;\text{of}\;\text{visceral}\;\text{fat}\;(\%)=\frac{\text{TWVF}}{\text{BW}}\times 100\%$$

TWVF is the abbreviation of the total weight of the visceral fat, which is the fat tissue collected from the previous tissues, and BW is the abbreviation of the bodyweight of the mouse. The unit of the weight in the formulae (4) is in grams (g).

### Measurement of Organ Weight

C57BL/6 mice were euthanized at the 22^nd^ week through exposure to carbon dioxide. The internal organs, such as the heart, liver, lungs, kidneys, spleen, and testicles, were dissected and collected from the sacrificed mice. The tissue weight was measured using an electronic scale and the tissue then immersed in 4% paraformaldehyde for fixation for subsequent histological sectioning (26, 27).

### Serological Analysis and Blood Element Analysis

C57BL/6 mice were sacrificed, and whole blood was collected *via* cardiac puncture. Serum was collected using Microtainer Serum Separator Tubes without any anticoagulant (or Eppendorf tube). The tube was then left upright for 30 min at room temperature and the sample centrifuged at 1,500*g* for 10 min at 4°C. Serological and blood element analyses were completed by the Laboratory Animal Center, College of Medicine, National Taiwan University.

### Hematoxylin and Eosin Stains

Hematoxylin and eosin stains (HE Stains) are used in histology and they are functions to recognize different types tissues and morphology. The organs harvested from sacrifice mouse are washed by 1× PBS solution twice and fixed with 4% paraformaldehyde solution at least 4 h at room temperature until tissue harden. The tissue was replaced 4% paraformaldehyde (30525-89-4,sigma-Aldrich, Inc.) solution with 70% ethanol, 80% ethanol, 95% ethanol, and 100% ethanol, and then tissues were embedded with paraffin as block. The paraffin block with organs is cut 4‐μm‐thick sections. The paraffin sections stained with hematoxylin about 10 min and eosin dye staining 2 min at room temperature. At least, the stained section mounted on a clear glass slide and observed by microscopy.

### Statistical Methods

Data are expressed as mean ± standard deviation of at least three repetitions. Statistical analyses were performed by one-way ANOVA and t-test analysis of the variance test. The results were considered signification when the p-value was < 0.05.

## Results

### Cell Viability and Cytotoxicity

**Figure 1:**
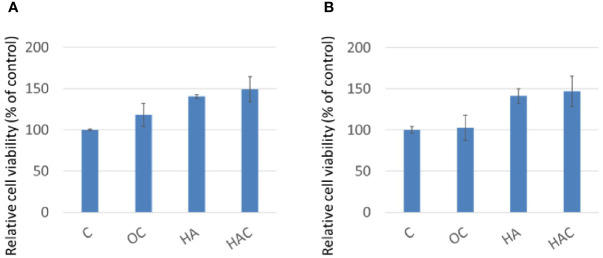
WST-1 assay shows cell viability at **(A)** day one and **(B)** day three. The results are expressed as mean ± standard deviation (SD) and the means of three independent experiments (n = 6), statistical analyses were performed by t-test analysis.

WST-1 assay was used to evaluate the cell viability, following the ISO-10993 regulation. As shown in **Figure 1**, the control group (C) was defined as 100%, where the cell viability for groups OC, HA, and HAC were all higher than that for the control group even on day one or day three, as shown in **Figures 1A, B**, respectively.

**Figure 2:**
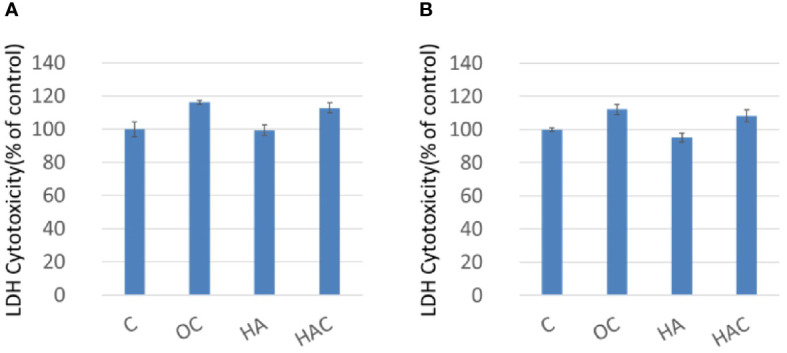
LDH assay shows cytotoxicity at **(A)** day one and **(B)** day three. The results are expressed as mean ± standard deviation (SD) and the means of three independent experiments (n = 6), statistical analyses were performed by t-test analysis.

The cytotoxicity was measured by LDH assay (**Figure 2**). The control group (C) was also defined as 100%. There was a significant difference in cytotoxicity for all the test groups on day one and day three as shown in **Figures 2A, B**, respectively.

As LDH assay (**Figure 2A**) shows that Clenbuterol probably still cytotoxic; however, group HAC is less toxic. In day one, the P value of group OC is 0.07500037, and the P value of HAC is 0.056150; In day three, the P value of group OC is 0.072115, and the P value of HAC is 0.055347. From a purely statistical perspective, the data demonstrates that there is no significant difference between control group and HAC. However, because the P value is actually very close to 0.05, there is still some room for further discussions.

From the results of the WST-1 and LDH analyses, we could tell that all the test groups kept 3T3-L1 in high cellular viability and was little toxicity to the 3T3-L1 cells.

### Oil-Red-O

**Figure 3:**
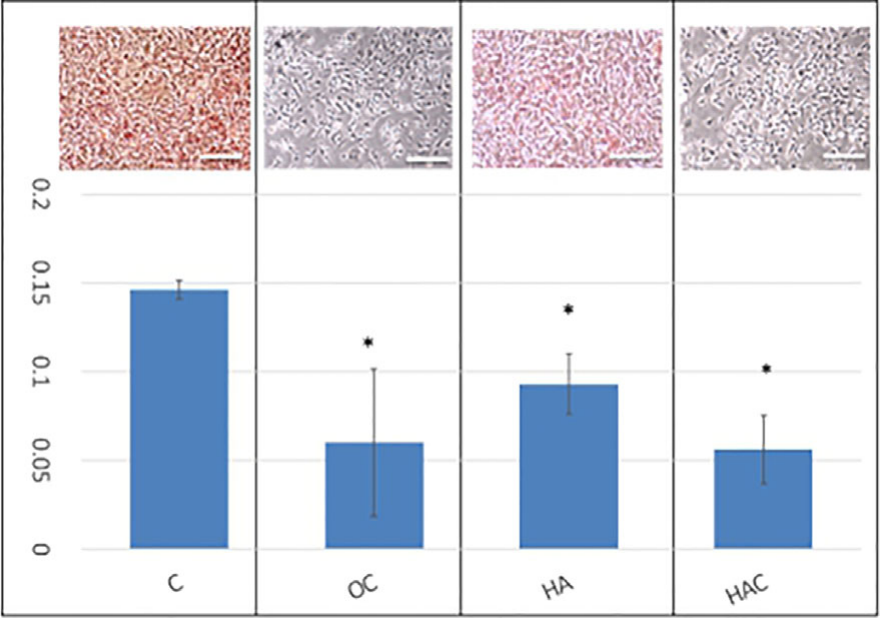
Oil-Red-O staining shows the tendency of lipogenesis. The upper part was the Oil-Red-O stain, and the lower part is the qualification based on the OD 520 nm. The results are expressed as mean ± standard deviation (SD) and the means of 3 independent experiments (n = 6), statistical analyses were performed by one-way ANOVA analysis. (*means p < 0.01).

Oil-Red-O is used for the staining of lipid droplets and lipid-containing vacuoles in cells. In this study, it was used because of the tendency of lipogenesis in 3T3-L1 cells, as shown in **Figure 3**. **Figure 3** was divided into two parts, where the upper part was the Oil-Red-O stain, and the lower part is the qualification based on the OD value. The control group and HA group showed a positive stain and a light stain in Oil-Red-O, respectively, as shown in the upper part of **Figure 3**. On the contrary, the OC group and HAC group displayed a negative stain in Oil-Red-O. In the qualification data, the OD value of the control group, OC group, HA group, and HAC group were 0.15, 0.06, 0.09, and 0.06, respectively.

The results of the Oil-Red-O analysis showed that OC and HAC could effectively inhibit the lipogenesis of 3T3-L1 cells.

### The Measurement of Body Weight

**Figure 4:**
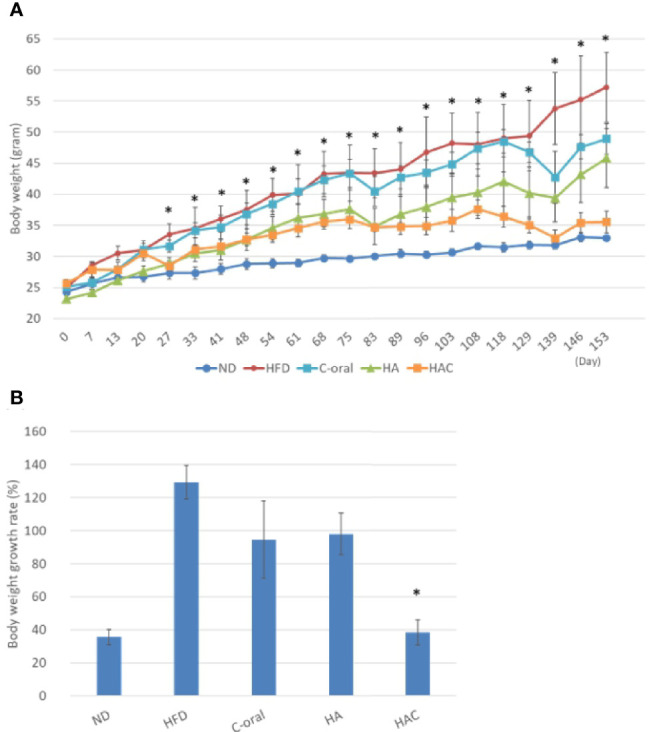
analysis of **(A)** the mouse body weights in the 22-week experimental period, where the arrows are the time to receive injection. **(B)** the body weight growth rate of the mice during the 22 weeks. Statistical analyses were performed by one-way ANOVA analysis. (*p < 0.05, n = 5).

**Figure 4** showed the bodyweight of the test animal in the experimental period for 22 weeks using one measurement a week. The body weight for the mice was increasing weekly (**Figure 4A**). However, it showed a different growth rate for each group (**Figure 4B**). The growth rate of body weight for 22 weeks in the normal diet (ND), high-fat diet (HFD), C-oral, HA, HAC groups were 136%, 229%, 195%, 198% and 138%, respectively; where it showed that the ND and HAC groups had the lowest growth rate and showed no statistical difference.

We believe that HAC by a single monthly controlled-release intra-adipose injection could reduce obesity, even the mice were fed a high-fat diet.

### Body Fat Analysis

**Figure 5:**
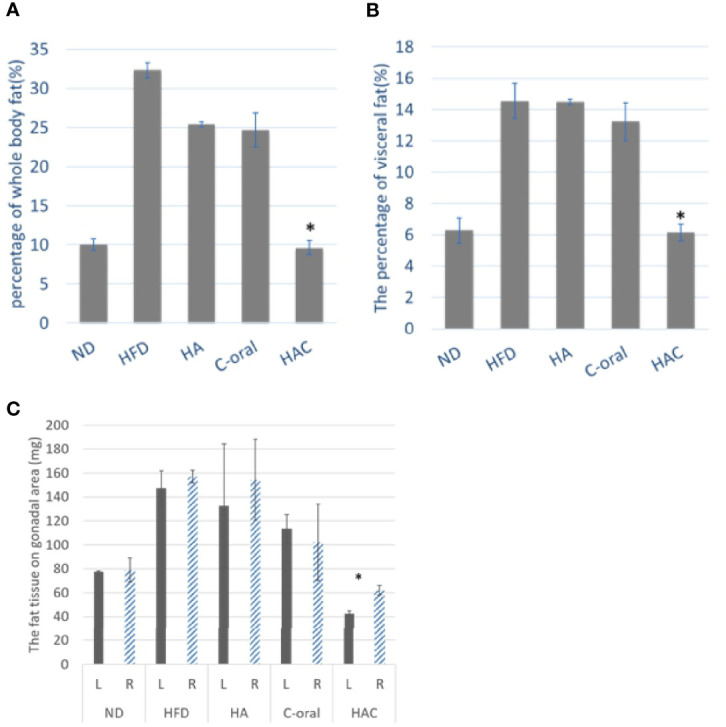
the mouse fat tissue analysis of **(A)** the total body fat, **(B)** the percentage of visceral fat, and **(C)** The fat tissue on the gonadal area, on week 22. Statistical analyses were performed by one-way ANOVA(a)(b) and t-test(c) analysis. (*p < 0.05, n = 5).

**Figure 5A** was the total body fat at the end of the experiment. The result was in agreement with that of body weight, where the mice receiving a normal diet and HAC had the lowest value in total body fat of 10.01% and 9.64%, respectively, without significant difference. The total body fat of mice under high-fat diet treatment was as high as 32.33%. There was no statistical difference in the HA and C-oral groups (25.37% and 24.68%, respectively).

**Figure 5B** was the measurement of the percentage of visceral fat. The results were similar to the whole-body fat. The normal diet (ND) and HAC groups showed the lowest values of 6.28% and 6.12%, respectively, with no significant difference. The mice with high-fat diet treatment had the highest value of 14.55%. There was no statistical difference in the HA and C-oral group (14.49% and 13.23%, respectively).

The fat tissue in the gonadal area was shown in **Figure 5C**. The injection was performed on the left side of the gonad. There was no significant difference in fat tissue between the left side and the right side of the gonadal adipose tissue area of the group on a normal diet, high-fat diet, HA, or C-oral. In contrast, the fat tissue showed a significant difference on the left and the right of the gonadal adipose tissue area of the HAC group. Additionally, the HAC group showed the lowest value on gonadal fat of 42.5 mg and 62.0 mg for the left side and right side, respectively.

The results of the measurement of whole-body fat and visceral fat showed that the mice that were given intra-adipose injections and a high-fat diet were effectively able to inhibit lipogenesis.

### MRI Analysis

**Figure 6:**
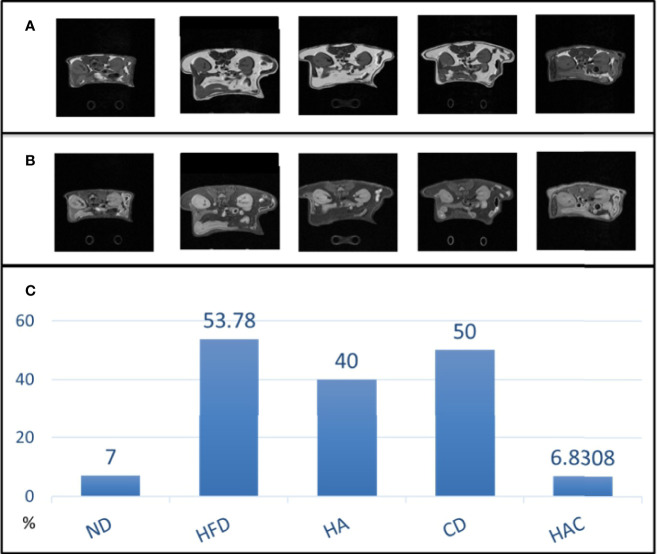
MRI imaging with **(A)** adipose mode, **(B)** water mode and **(C)** overall fat ratio.

The abdominal fat was analyzed by MRI imaging in adipose and water modes as shown in **Figures 6A, B**, respectively. The overall fat ratio is recorded in **Figure 6C**, where the abdominal fat ratio in the normal diet and HAC groups were 7% and 6.83% by area, respectively, without a statistically significant difference. The group HFD showed the highest value, 53.78%. The abdominal fat ratio by MRI imaging was used to reflect the visceral fat tissue.

### Organ Weight Analysis

The evaluation of organ weights in toxicology is an integral component in the assessment of pharmaceuticals and chemicals. The Society of Toxicologic Pathology (STP) has recommended weighing organs in GLP general toxicology studies lasting from 7 days to 1 year. In this study, the organ weights of the experimental mice were recorded at the end of the 22^nd^ week, as seen in **Table 2**. Interestingly, the organ weight showed no statistical difference for most of the groups. Among the test groups, the weights of testicles and kidneys in the C-oral group showed abnormal highs. We believe that C-oral might be toxic to the kidney and testes.

**Table 2:** The organs weight for all the test groups (*p < 0.05).

	Heart	Lung	Spleen	Liver	Kidney	Testicle
ND	186.0 ± 22.5	284.5 ± 19.0	80.4 ± 14.6	1485.8 ± 160.2	519.0 ± 35.8	191.2 ± 9.6
HFD	228.5 ± 3.9	273.3 ± 86.3	114.0 ± 3.3	1571.0 ± 112.85	612.3 ± 11.0	184.4 ± 4.9
C-oral	185.4 ± 13.0	295.7 ± 19.5	91.5 ± 6.15	1542.4 ± 66.9	989.5 ± 130.9*	560.0 ± 192.5*
HA	162.0 ± 18.0	323.1 ± 55.2	101.3 ± 12.5	1444.5 ± 296.5	486.6 ± 32.2	215.8 ± 14.0
HAC	203.5 ± 4.7	317.2 ± 20.5	109.9 ± 9.5	156.4 ± 95.3	493.2 ± 5.7	189.6 ± 4.8

The HE stain for all the harvested organs showed no significant difference for all test groups, as shown in **Supplementary Figure 1**.

### The Blood Element and Serological Analysis

Whole blood element analysis is a practical tool that assists in determining deficiencies, excesses, and imbalances of essential elements as well as recent or ongoing exposure to specific toxic substances. The analysis of blood elements in WBC, NE, LY, MO, EO, BA, RBC, HGB, HCT, and MCV are shown in **Table 3**, where the results showed no abnormal values for all test groups.

**Table 3:** The blood element analysis for all the test groups (*p < 0.05).

	ND	HFD	C-oral	HA	HAC
WBC (10^3^/μl)	7.22 ± 2.08	10.2 ± 3.37	12.11 ± 3.03	11.26 ± 4.66	8.51 ± 0.92
NE%	24.03 ± 13.14	16.53 ± 2.68	27.04 ± 15.99	26.04 ± 7.68	32.13 ± 4.29
LY%	61.36 ± 7.09	64.28 ± 22.08	60.98 ± 15.29	55.23 ± 3.04	62.31 ± 5.38
MO%	0.42 ± 0.32	4.35 ± 3.44	2.99 ± 1.57	1.44 ± 1.39	0.56 ± 0.25
EO%	0.34 ± 0.2	0.7 ± 0.11	0.71 ± 0.24	0.4 ± 0.09	0.42 ± 0.25
BA%	0.02 ± 0	0.28 ± 0.21	0.25 ± 0.13	0.85 ± 0.06	0.16 ± 0.09
RBC (10^6^/μl)	10.45 ± 2.09	8.24 ± 0.04	10.41 ± 2.34	13.11 ± 3.32	10.53 ± 1.22
HGB (g/dl)	15.53 ± 2.64	18.15 ± 2.25	14.73 ± 0.82	14.77 ± 1.25	14.65 ± 2.02
HCT(%)	45.77 ± 1.51	48.4 ± 1.7	42.5 ± 2.13	45.68 ± 6.16	40.94 ± 2.44
MCV(fl)	54.86 ± 17.56	60.44 ± 2.77	52.72 ± 31.97	53.46 ± 12.7	48.2 ± 11.1
MCH (pg)	14.43 ± 1.32	16.62 ± 3.42	18.08 ± 1.02	16.06 ± 1.63	16.54 ± 5.15
MCHC (g/dl)	15.86 ± 2.57	25.09 ± 9.28	19.94 ± 9.84	19.71 ± 3.6	17.3 ± 1.26
RDW(%)	20.52 ± 4.84	16.74 ± 2.94	22.35 ± 5.64	20.59 ± 4.07	20.63 ± 4.44
PLT(10^3^/μl)	284.63 ± 137.2	201.75 ± 39.45	298 ± 57.06	538.73 ± 184.87	281.13 ± 88.58
MPV (fl) (10^6^/μl)	5.66 ± 0.2	6.99 ± 0.32	5.66 ± 0.59	3.15 ± 0.66	4.07 ± 2.42

The serological parameters are important indexes to analyze toxicity during the development of medical substances. In the study, ALT and AST were used to examine liver toxicity; BUN and Crea were to evaluate kidney function; and GLU, HDL, Na^+^, and TG were used to check overall blood sugar and blood lipidemic. The results are shown in **Tables 4** and **5**. In the C-oral group, ALT and AST were abnormally high in blood level, and the Na^+^ blood level was also much higher than the normal group. There were no significant differences for most of the test groups.

**Table 4:** The serological analysis for all the groups (*p < 0.05).

	ALT	AST	BUN	Crea
	U/liter	U/liter	mg/dl	mg/dl
ND	38.7 ± 18.6	76.4 ± 49.0	15.4 ± 2.8	0.4
HFD	15.0 ± 5.1	63.1 ± 5.9	18.9 ± 6.4	0.1
C-oral	144.0 ± 7.5*	395.7 ± 128.3*	22.2 ± 6.0*	0.2
HA	89.3 ± 60.6	149.2 ± 15.0	16.8 ± 3.9	0.2
HAC	22.0 ± 11.2	54.6 ± 16.0	12.0 ± 2.5	0.1

**Table 5:** the serological analysis data on *in vivo* study (*p < 0.05).

	GLU	HDL	Na+	TG
	mg/dl	mg/dl	mmol/liter	mg/dl
ND	133.69 ± 4.42	43 ± 2.16	111.83 ± 8.72	130.19 ± 15.29
HFD	189.14 ± 17.55	162.67 ± 27.35	141.67 ± 14.84	454.27 ± 84.34
C-oral	207.91 ± 19.29	100 ± 52.56	172.33 ± 63.56*	148.35 ± 17.8
HA	109.67 ± 8.59	49.8 ± 2.89	96.33 ± 11.95	260.86 ± 25.22
HAC	141.28 ± 10.96	70.9 ± 25.36	98.2 ± 11.92	217.33 ± 20.83

## Discussion

Clenbuterol is a steroid-like chemical that was initially developed to treat asthma in horses by relaxing the airways in the animals’ lungs. It stimulates both the heart and central nervous system and has a similar effect as epinephrine and amphetamines on the body. This increase will lead to a variety of effects, such as rapid fat burning so that Clenbuterol can be used as a weight-loss aid by increasing a person’s metabolism. As well as reducing body fat and body weight, it also retains both muscle mass and body strength at the same time. Many bodybuilders, performance athletes, and those wanting to lose weight currently use the drug (11, 28, 29).

In the *in-vitro* study, OC and HAC could effectively inhibit the lipogenesis of 3T3-L1 cells in Oil-Red-O analysis due to being Clenbuterol containing, as shown in **Figure 3**.

In the animal study, the mice receiving a high-fat diet and treated with Clenbuterol by oral administration (C-oral group) or injected by HAC regularly effectively reduced their whole body fat, body weight, visceral fat, and gonadal fat; even to a lower extent than the mice treated with a normal diet (ND group), as shown in **Figures 4**–**6**.

Interestingly, in **Figures 4**–**6** show that HA injection may be effective in anti-obesity. Currently there is no supportive reference about that HA can induce lipolysis; there is only reference about that HA induces the differentiation from 3T3L1 to adipocyte *in vivo* (20). Also, in this study the HA group demonstrates that both body weight and body fat decreased.

As the bio-safety *in-vitro* test, WST-1 and LDH were used to prove that the dose in all the test groups was not toxic to 3T3-L1 cells. In the animal study, we used the Clenbuterol dose at the established safety level using a controlled release of 2 µg – 10 µg per daily; the dose was far below the bodybuilder and athlete daily dose of 20 mg – 140 mg. The dose in our study by weight was 0.1 mg – 0.5 mg per kilogram. It was proved to be safe in the *in-vivo* test of chronic toxicity using the blood elements and serological analyses, as shown in **Tables 3**–**5**, respectively. A prior study showed that the oral intake of Clenbuterol had an increased volume and therapeutic effect on the Leydig cell of testicles (26). It was mentioned in prior research that hypernatremia caused by Clenbuterol might be the cause of increased kidney volume. In our study, the results corresponded with the previous studies, as seen in the abnormal increases observed in the C-oral group in AST/ALT, BUN/kidney-weight, Na^+^, and testicle weight. These were correlated to hepatoxicity, renal toxicity, hypernatremia, and testicular function.

Recently, body fat percentage (FATR) has been considered a more precise method to evaluate obesity status than BMI. Furthermore, most obesity occurs owing to abdominal adipose tissue accumulation in apple-shaped and pear-shaped body types. The gonadal fat tissue in mice is highly co-related to apple-shaped and pear-shaped body types caused by symmetrical growth (30). Instead of body weight control, the distribution of adipose tissue and FATR are now highly emphasized among anti-obesity groups (31). In this study, we injected HAC in the left gonad; it demonstrated lower weight and volume in the left gonadal fat tissue than in the right, as shown in **Figure 5C**.

The study examined the weight of visceral fat around the internal organs. The results are shown in **Figure 5B**. As is known, visceral fat is the body fat that is stored within the abdominal cavity and around some internal organs such as the liver, kidney, and intestines. Visceral fat is referred to as ‘active fat’ that plays a distinctive and potentially dangerous role affecting how our hormones function and actively increases the risk of severe health problems. Storing higher amounts of visceral fat is associated with increased risks of several health problems, including type 2 diabetes and stroke. In this study, the abdominal fat was analyzed using MRI imaging in adipose and water modes as shown in **Figures 6A, B**, respectively. The abdominal fat ratio in the normal diet and HAC groups had the lowest values among the test groups. The HFD group had the highest value (53.78%). The abdominal fat ratio using MRI imaging was generally used to reflect the visceral fat tissue.

The high-fat diet-induced obesity mouse model was commonly used to mimic the increasing availability of high-fat and high-density food in individuals’ daily lifestyles. The 60 kcal% fat diet-induced obesity mouse could be co-related to the unhealthy dietary habit of modern people. The mouse used in the study is in the sexually mature adolescent stage, similar to the age distribution of the largest population using diet drugs to fight obesity.

Clenbuterol, as a beta-receptor agonist, is inadvisable to co-administer with some beta-blockers such as cardiovascular disease drugs and anti-hypertensives. However, regulatory restrictions have not effectively stopped Clenbuterol abuse. We believe that changing the method of administration to reduce the toxicity level might be another approach toward the legal use of Clenbuterol. Improvement of the dosage and administration might be the possible way to prevent damage caused by fitness enthusiasts using Clenbuterol while pursuing efficient weight loss and muscular gains (12).

## Conclusion

This study developed a new and promising method to combat obesity through a single, monthly controlled-release intra-adipose injection of Clenbuterol-modified hyaluronic acid thermo-sensitive hydrogel. The newly developed Clenbuterol formula is effective not only in decreasing body weight and body fat content but also in inhibiting lipogenesis in harvested visceral tissue. It also reduces adipose tissue around the gonadal area. The side effects of the traditional oral administration of Clenbuterol were not observed in this study using HAC intra-adipose injection to achieve controlled release.

## Data Availability Statement

The raw data supporting the conclusions of this article will be made available by the authors, without undue reservation.

## Ethics Statement

The animal study was reviewed and approved by Institutional Animal Care and Use Committee, National Taiwan University, Taipei, Taiwan.

## Author Contributions

All authors listed have made a substantial, direct, and intellectual contribution to the work and approved it for publication.

## Funding

This study was supported by National Health Research Institutes, and Central Government S & T grant, Taiwan (108-0324-01-19-07).

## Conflict of Interest

The authors declare that the research was conducted in the absence of any commercial or financial relationships that could be construed as a potential conflict of interest.

## References

[1] Muuronen AT Taina M Hedman M Marttila J Kuusisto J Onatsu J. Increased visceral adipose tissue as a potential risk factor in patients with embolic stroke of undetermined source (ESUS). PloS One (2015) 10(3):e0120598. 10.1371/journal.pone.0120598 25756793 PMC4354901

[2] Naja F Hwalla N Itani L Karam S Sibai AM Nasreddine L. A Western dietary pattern is associated with overweight and obesity in a national sample of Lebanese adolescents (13-19 years): a cross-sectional study. Br J Nutr (2015) 114(11):1909–19. 10.1017/S0007114515003657 PMC4635384 26431469

[3] Anders S Schroeter C. The impact of nutritional supplement intake on diet behavior and obesity outcomes. PloS One (2017) 12(10):e0185258. 10.1371/journal.pone.0185258 28991921 PMC5633155

[4] Kanauchi O Deuchi K Imasato Y Shizukuishi M Kobayashi E. Mechanism for the inhibition of fat digestion by chitosan and for the synergistic effect of ascorbate. Biosci Biotechnol Biochem (1995) 59(5):786–90. 10.1271/bbb.59.786 7787293

[5] Zhang W-S An Pan LY Cai Y-Y Liu B-L Li P Qi L-W. American Ginseng and Asian Ginseng Intervention in Diet-Induced Obese Mice: Metabolomics Reveals Distinct Metabolic Profiles. Am J Chin Med (2017) 4:787–801. 10.1142/S0192415X19500411 31091973

[6] Gadde KM Martin CK Berthoud HR Heymsfield SB. Obesity: Pathophysiology and Management. J Am Coll Cardiol (2018) 71(1):69–84. 10.1016/j.jacc.2017.11.011 29301630 PMC7958889

[7] Iannelli A Dainese R Piche T Facchiano E Gugenheim J. Laparoscopic sleeve gastrectomy for morbid obesity. World J Gastroenterol (2008) 14(6):821–7. 10.3748/wjg.14.821 PMC2687048 18240338

[8] Santoro S Malzoni CE Velhote MC Milleo FQ Santo MA Klajner S. Digestive Adaptation with Intestinal Reserve: a neuroendocrine-based operation for morbid obesity. Obes Surg (2006) 16(10):1371–9. 10.1381/096089206778663841 17059749

[9] Mitchell GA Dunnavan G. Illegal use of beta-adrenergic agonists in the United States. J Anim Sci (1998) 76(1):208–11. 10.2527/1998.761208x 9464900

[10] Liu J Zeng L Li Z Gao F Huang X Li F. Solid substrate-room temperature phosphorimetry for the determination of residual clenbuterol hydrochloride based on the catalysis of sodium periodate oxidizing eosine Y. Anal Chim Acta (2009) 638(1):69–74. 10.1016/j.aca.2009.02.007 19298881

[11] Kintz P Gheddar L Ameline A Dumestre-Toulet V Verschoore M Comte J. Complete Post-mortem Investigations in a Death Involving Clenbuterol After Long-term Abuse. J Anal Toxicol (2019) 43(8):660–5. 10.1093/jat/bkz058 31436794

[12] Ip EJ Doroudgar S Lau B Barnett MJ. Anabolic steroid users’ misuse of non-traditional prescription drugs. Res Soc Adm Pharm (2019) 15(8):949–52. 10.1016/j.sapharm.2018.07.003 31303195

[13] Selyanin MA Boykov PY Khabarov VN Polyak F. The Biological Role of Hyaluronic Acid. In: Hyaluronic Acid. (2015). p. 9–75.

[14] Valachova K Volpi N Stern R Soltes L. Hyaluronan in Medical Practice. Curr Med Chem (2016) 23(31):3607–17. 10.2174/0929867323666160824162133 27554806

[15] Su WY Chen YC Lin FH. Injectable oxidized hyaluronic acid/adipic acid dihydrazide hydrogel for nucleus pulposus regeneration. Acta Biomater (2010) 6(8):3044–55. 10.1016/j.actbio.2010.02.037 20193782

[16] Hu MH Yang KC Sun YH Chen YC Yang SH Lin FH. In situ forming oxidised hyaluronic acid/adipic acid dihydrazide hydrogel for prevention of epidural fibrosis after laminectomy. Eur Cell Mater (2017) 34:307–20. 10.22203/eCM.v034a19 29130237

[17] Chen YC Su WY Yang SH Gefen A Lin FH. In situ forming hydrogels composed of oxidized high molecular weight hyaluronic acid and gelatin for nucleus pulposus regeneration. Acta Biomater (2013) 9(2):5181–93. 10.1016/j.actbio.2012.09.039 23041783

[18] Shoham N Sasson AL Lin FH Benayahu D Haj-Ali R Gefen A. The mechanics of hyaluronic acid/adipic acid dihydrazide hydrogel: towards developing a vessel for delivery of preadipocytes to native tissues. J Mech Behav BioMed Mater (2013) 28:320–31. 10.1016/j.jmbbm.2013.08.009 24021174

[19] Liu J Liu Z-b Huang Q Lin C-Q Lin X. Highly Sensitive Fluorescent Probe for Clenbuterol Hydrochloride Detection Based on its Catalytic Oxidation of Eosine Y by NaIO4. J Fluorescence (2014) 24(5):1495–501. 10.1007/s10895-014-1435-7 25155629

[20] Ji E Jung MY Park JH Kim S Seo CR Park KW. Inhibition of adipogenesis in 3T3-L1 cells and suppression of abdominal fat accumulation in high-fat diet-feeding C57BL/6J mice after downregulation of hyaluronic acid. Int J Obesity (2014) 38(8):1035–43. 10.1038/ijo.2013.202 24173405

[21] Wang CY Liao JK. A mouse model of diet-induced obesity and insulin resistance. Methods Mol Biol (2012) 821:421–33. 10.1007/978-1-61779-430-8_27 PMC3807094 22125082

[22] Yang Y Smith DL Jr. Keating KD Allison DB Nagy TR. Variations in body weight, food intake and body composition after long-term high-fat diet feeding in C57BL/6J mice. Obesity (Silver Spring) (2014) 22(10):2147–55. 10.1002/oby.20811 PMC4180788 24942674

[23] Hong J Stubbins RE Smith RR Harvey AE Nunez NP. Differential susceptibility to obesity between male, female and ovariectomized female mice. Nutr J (2009) 8:11. 10.1186/1475-2891-8-11 19220919 PMC2650703

[24] Simchick G Yin A Yin H Zhao Q. Fat spectral modeling on triglyceride composition quantification using chemical shift encoded magnetic resonance imaging. Magn Reson Imaging (2018) 52:84–93. 10.1016/j.mri.2018.06.012 29928937 PMC6537901

[25] Johnson DH Narayan S Wilson DL Flask CA. Body composition analysis of obesity and hepatic steatosis in mice by relaxation compensated fat fraction (RCFF) MRI. J Magn Reson Imaging (2012) 35(4):837–43. 10.1002/jmri.23508 PMC3288219 22095745

[26] Lee MR Kim JE Choi JY Park JJ Kim HR Song BR. Anti-obesity effect in high-fat-diet-induced obese C57BL/6 mice: Study of a novel extract from mulberry (Morus alba) leaves fermented with Cordyceps militaris. Exp Ther Med (2019) 17(3):2185–93. 10.3892/etm.2019.7191 PMC6395968 30867704

[27] Betzel B Cooiman MI Aarts EO Janssen IMC Wahab PJ Groenen MJM. Clinical follow-up on weight loss, glycemic control, and safety aspects of 24 months of duodenal-jejunal bypass liner implantation. Surg Endosc (2019) 209–15. 10.1007/s00464-019-06752-8 PMC6946747 30877567

[28] Schifano F Chiappini S Corkery JM Guirguis A. Abuse of Prescription Drugs in the Context of Novel Psychoactive Substances (NPS): A Systematic Review. Brain Sci (2018) 8(4):73. 10.3390/brainsci8040073 PMC5924409 29690558

[29] Li C Adhikari BK Gao L Zhang S Liu Q Wang Y. Performance-Enhancing Drugs Abuse Caused Cardiomyopathy and Acute Hepatic Injury in a Young Bodybuilder. Am J Mens Health (2018) 12(5):1700–4. 10.1177/1557988318783504 PMC6142118 29926766

[30] Karastergiou K Smith SR Greenberg AS Fried SK. Sex differences in human adipose tissues – the biology of pear shape. Biol Sex Dif (2012) 8(4):73. 10.1186/2042-6410-3-13 PMC3411490 22651247

[31] Swainson MG Batterham AM Tsakirides C Rutherford ZH Hind K. Prediction of whole-body fat percentage and visceral adipose tissue mass from five anthropometric variables. PloS One (2017) 12(5):e0177175. 10.1371/journal.pone.0177175 28493988 PMC5426673

