# Supplementary material for: Oxidized Hyaluronic Acid Hydrogels as a Carrier for Constant-Release Clenbuterol Against High-Fat Diet-Induced Obesity in Mice
Source: Front Endocrinol (Lausanne). 2021 Mar 12;12:572690. doi: 10.3389/fendo.2021.572690 (PMC7996091; doi:10.3389/fendo.2021.572690)
Supplement: Supplementary file 1 [file DataSheet_1.docx]

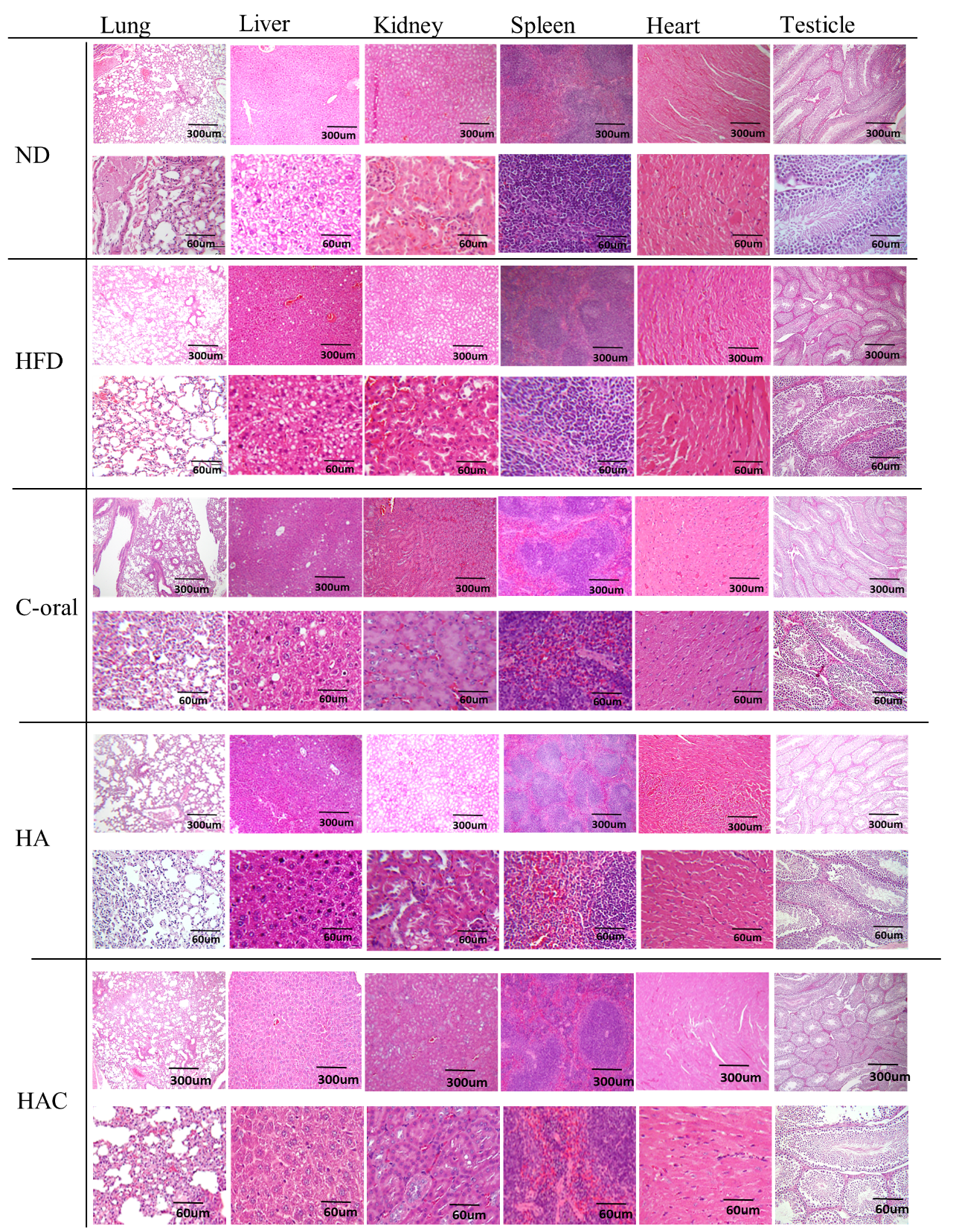


Supplementary fig. 1 The histology analysis of hematoxylin eosin staining

Supplementary Table 1 the physiological data for groups on *in vivo* study

|  | | Resp. (Breathe/m) | | | PR (bpm) |  |
| --- | --- | --- | --- | --- | --- | --- |
| ND | 18-20 | |  | 210-294 | | |
| HFD | 30-38 | |  | 300-340 | | |
| HA | 21-27 | |  | 370-380 | | |
| C-oral | 14-18 | |  | 228-262 | | |
| HAC | 15-18 | |  | 292-310 | | |

Supplementary Table 2 The anatomy data of fat weight for groups on *in vivo* study

|  | ND | HFD | C-oral | HA | HAC |
| --- | --- | --- | --- | --- | --- |
| Body weight (g) | 32.3 ± 0.9 | 49.9± 0.3 | 47.1 ± 0.9 | 46.9 ± 2.8 | 36.1 ± 0.1 |
| Fat weight of gonadal (mg) | 507.1 ± 106.5 | 386.8± 76.3 | 551.0 ± 40.0 | 493.5 ± 55.6 | 186.3 ± 27.6 |
| Fat weight of gonadal subcutaneous (mg) | 156.5 ± 5.9 | 304.5 ± 9.8 | 281.3 ± 17.6 | 287.0 ± 42.7 | 104.6 ± 9.8 |
| Fat weight of Perirenal (mg) | 447.3 ± 77.5 | 1755.1± 108.2 | 1868.9 ± 141.1 | 1683.9 ± 97.2 | 369.5 ± 11.7 |
| Fat weight of abdomen (visceral) (mg) | 810.2 ± 54.7 | 3470.5 ± 231.9 | 3487.3 ± 156.6 | 2987.8 ± 95.3 | 1346.7± 8.6 |
| Fat weight of abdomen subcutaneous (mg) | 643.7 ± 17.2 | 3270.3 ± 213.4 | 2625.1± 163.6 | 2866.8 ± 177.2 | 554.9 ± 67.1 |
| Fat weight of mesentery (mg) | 261.8 ± 126.3 | 1653.5 ± 272.0 | 560.9 ± 16.9 | 1632.5 ± 120.0 | 305.4 ± 23.6 |
| Fat weight of anterior and scapular (mg) | 405.4 ± 14.3 | 5301.6 ± 154.3 | 2486.1 ± 217.9 | 1947.2 ± 114.5 | 607.7 ± 67.1 |
| Body fat percentage (FATR) % | 10.02 | 32.33 | 24.68 | 25.37 | 9.64 |
| Visceral fat percentage % | 6.28 | 14.55 | 13.23 | 14.49 | 6.12 |

(weeks-old)

Supplementary fig. 2 The food intake data accumulation each cage (5 mice)
